# Two New Sandwich-Type Polyoxomolybdates Functionalized with Diphosphonates: Efficient and Selective Oxidation of Sulfides to Sulfones

**DOI:** 10.3390/ma10101173

**Published:** 2017-10-13

**Authors:** Qiaofei Xu, Xiaopeng Sun, Feng Hu, Rong Wan, Vikram Singh, Pengtao Ma, Jingyang Niu, Jingping Wang

**Affiliations:** Henan Key Laboratory of Polyoxometalate Chemistry, Institute of Molecule and Crystal Engineering, College of Chemistry and Chemical Engineering, Henan University, Kaifeng 475004, China; xqf199408a@163.com (Q.X.); sunxiaopeng2013@163.com (X.S.); huf17@mails.tsinghua.edu.cn (F.H.); wanrong1992@163.com (R.W.); singhvikram001@gmail.com (V.S.); mpt@henu.edu.cn (P.M.)

**Keywords:** polyoxomolybdates, catalyst, oxidation of sulfides

## Abstract

Two sandwich-type polyoxomolybdates Na_8_[MO_2_{Mo_2_O_5_(O_3_PCH_3_C(O)PO_3_)}_2_] (M = Ni^2+^ (**1**); Co^2+^ (**2**)) were synthesized by one-pot reaction of Na_2_HPMo_12_O_40_·14H_2_O, 1-hydroxy ethidene diphosphonic acid (HEDP=HOC(CH_3_)(PO_3_H_2_)_2_), and (1) NiCl_2_/CoCl_2_ (2). Compounds **1** and **2** were characterized by single crystal X-ray analysis, X-ray powder diffraction (XRPD), IR spectroscopy, ^31^P NMR spectra, UV-vis spectroscopy, and thermogravimetric analyses (TGA). Structural analysis reveals that **1** and **2** exhibit similar centrosymmetric structure, which consists of one transition metal (TM) ion sandwiched by two same subunits {Mo_2_O_5_(O_3_PCH_3_C(O)PO_3_)}. The clusters 1 and 2 show efficient catalytic activities for oxidation of thioanisole. Moreover, they are catalytically selective for oxidizing thioanisole. Both resuable polyoxomolybdates **1** and **2** catalysts show good thermo- and hydrolytic stability. It is noted that compound **1** shows outstanding catalytic activity for oxidation of various sulfides to corresponding sulfones with 93–100% selectivity at 97–100% conversion in one hour under mild conditions, which is potentially valuable to the removal of organic sulfides.

## 1. Introduction

Selective oxidation of sulfides are important transformations in synthetic organic chemistry and active area of research in industries, as the corresponding oxidation products i.e., sulfoxides and sulfones play vital roles in the synthesis of fine chemicals, pharmaceuticals, oxotransfer reagents, biologically active molecules, and ligands of chiral catalysts [1,2,3,4,5,6,7]. In addition, oxidative desulfurization is also a critical process for desulfurization of fuel oil, which has been consistently studied for its high efficiency [8,9]. Hence, considerable research efforts have been focused on these reactions. Progressively, UHP (Hydrogen peroxide–Urea adduct) [10], NaClO [11], NaIO_4_ [12], and oxone [13], etc., have been developed along with the previously explored biological enzymes and transition metal based (TMs) catalysts that are being employed in the process [14,15,16,17,18]. Nevertheless, some problematic disadvantages in the aforementioned systems cannot be overlooked when considering the environmental and economic benefits; it is thus an imperative to use hydrogen peroxide as a first-rate oxidant instead of other classical toxic waste-producing oxidants [19]. Moreover, the excellent catalysts should be highly efficient, selective, robust, and have good recyclability [20].

As it is known, polyoxometalates (POMs) have attracted more and more interest because they possess unique properties in catalysis, magnetism, molecular electronics, biology, and in pharmacy, etc. [21,22,23,24,25,26,27,28]. The nucleophilic surface-oxygen-enriched polyanions have outstanding redox features, which have led POMs to be an excellent candidate for catalytic oxidization of organic compounds. Importantly, it is well known that POMs show remarkable thermal, hydrolytic, and oxidative stability [29], which implies them as a best choice as catalytic materials for oxidation systems. Recently, Yang et al. [30] and Mizuno et al. [31] have employed POMs as splendid catalysts for the transformations of sulfides. In addition, POMs supported on polymer and mesoporous silica have shown great catalytic activities for various organic reactions [32,33]. Recently, 1-hydroxy ethidene diphosphonic acid (HEDP=HOC(CH_3_)(PO_3_H_2_)_2_), a kind of diphosphonates [34], has been introduced to obtain organic functionalized POMs.

It is to be noted that HEDP shows a similar framework to the pyrophosphate (P_2_O_7_^4−^) where the center oxygen atom is substituted by a C atom producing P–C–P backbone, and providing more stability in solution than all-inorganic pyrophosphates (P_2_O_7_^4−^) [35]. Taken these facts together, it is possible to use HEDP as a building block to synthesize robust POMs that could be used as an efficient catalyst. As the structure of POMs can be designed at atomic or molecular level, it is wonderful to introduce HEDP and TMs into POMs to generate new excellent catalysts for oxidizing sulfides. We herein, synthesized two new sandwich-type TMs-containing polyoxomolybdates functionalized with HEDP: Na_8_[NiO_2_{Mo_2_O_5_(O_3_PCH_3_C(O)PO_3_)}_2_]·26H_2_O (**1**) and Na_8_[CoO_2_{Mo_2_O_5_(O_3_PCH_3_C(O)PO_3_)}_2_]·24H_2_O (**2**), where TMs and HEDP are successfully taken together in their molecular frameworks. Both polyoxomolybdates **1** and **2** exhibit similar catalytic activity for thioanisole oxidization, preferably **1** finds its selectivity for oxidizing thioethers. We also investigated the detail studies on account of its better catalysis, which covers its preferable recyclability, desirable catalytic activity, and selectivity for oxidizing sulfides to sulfones under mild conditions.

## 2. Results and Discussion

### 2.1. Structure Description

**Figure 1:**
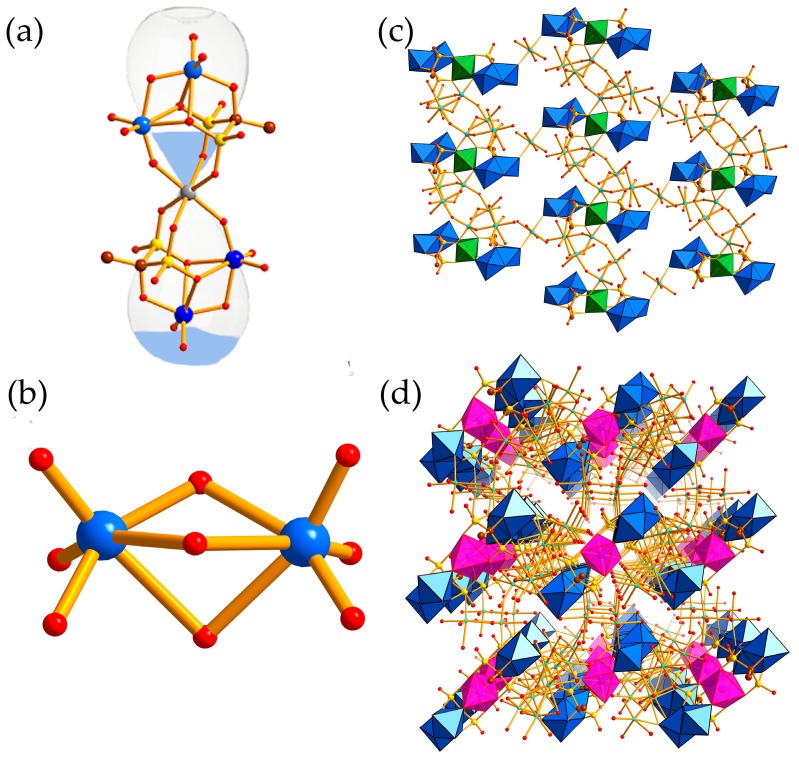
(**a**) polyhedral/ball-and-stick view of the monomer of compound **1** and **2**; (**b**) Ball-and-stick view of {Mo^VI^_2_O_5_} moieties; (**c**) the two-dimensional (2D) planar structure of compound **1**; (**d**) the three-dimensional (3D) structure of compound **2**; (MoO_6_/Mo: blue, tetrahedron of P/P: yellow, Co: amaranth, Ni: green, C: brown, O: red, Na: cyan-blue, Ni/Co: gray.)

X-ray single crystal diffraction indicate that **1** crystallizes in the triclinic space group *P*-1 and **2** crystallizes in the monoclinic space group *C*2/*c* (Table 1). They are centrosymmetric and isomorphic with the common polyanions ([MO_2_{Mo_2_O_5_(O_3_PCH_3_C(O)PO_3_)}_2_]^8−^ (M = Ni^2+^ and Co^2+^), Figure 1a). One polyanion consists of four Mo atoms, two HEDP ligands, ten *μ*_2_-O groups, four *μ*_3_-O, and twelve terminal O groups. Interestingly, transition metal ion is sandwiched by two {Mo_2_O_5_(O_3_PCH_3_C(O)PO_3_)} subunits generating the polyanion, whereas, HEDP and {Mo_2_O_5_} construct the subunits via the C–O_Mo_ and P–O_Mo_ linkers. Notably, the face-sharing of two {MoO_6_} octahedrons of {Mo^VI^_2_O_5_} moieties (Figure 1b) in **1** and **2** are much different from the reported {Mo^VI^_2_O_4_}, which are in an edge-sharing fashion [36]. The lengths of Mo–O bonds are in the range of 1.7128 (31)–2.4232 (28) Å and 1.7104 (36)–2.3659 (31) Å in **1** and **2**, respectively. Beside this, the six-coordinated M^II^ cation adopts octahedral geometry, and bonding to two same asymmetric units by six bridging O atoms that forms structural units. Furthermore, in the polyanions, four Mo atoms and the TM atom are located in the paper, which is also the symmetry plane of the monomer. Additionally, there is also a plane including four P atoms and the TM atom, which is almost perpendicular to the paper with the dihedral angles of 87.190 (24)° and 89.340 (18)° for **1** and **2**, respectively.

In addition, it is noted that there is a hexanuclear {Na_6_} cluster in **1** (Figure S3a). Furthermore, the {Na_6_} clusters and monomeric units generate one-dimensional (1D) chain (Figure S3c) by their interconnection: six-coordinate sodium ions bonded to nickel atoms and P atoms, and the neighboring 1D chains are linked by single Na^+^ resulting in two-dimensional (2D) plane (Figure 1c). Unlike **1**, hexanuclear {Na_6_} clusters also exist in compound **2**, but their connection modes are different: sodium clusters in compound **2** formed 1D chains (Figure S4b) by μ_2_-O atoms, which are attached to monomers by Mo–O_Na_ and P–O_Na_ bonds to construct 2D flat (Figure S4d). These 2D flats are linked by dimers of sodium ions, generating the three-dimensional (3D) structure of compound **2** (Figure 1d).

### 2.2. Catalytic Activity

Initially, the oxidation of thioanisole by 30% H_2_O_2_ was carried out in the presence of **1**. The results of oxidizing thioanisole under different conditions were summarized in Table 2.

#### 2.2.1. The Exploration of Optimal Conditions

● Effect of amount of catalyst

As shown in Table 2 (entry 1–4), there is little conversion of thioanisole with no addition of **1**. On increasing the amount of the catalyst from 8 to 17 mg, the conversion has promoted from 90% to 96% and corresponding change in selectivity increases from 80% to 89%. The further addition of catalyst up to 25 mg, a little increase in conversion rate (98%) and selectivity (94%) were observed. In this process, increase in catalytic dosage may produce more active species that factors the transformation from methyl phenyl sulfide to methyl phenyl sulfone.

● Effect of temperature

As can be seen from Table 2 (entries 4–6), the effect of temperature was studied to optimize the reaction condition. The catalytic activity of catalyst is 63% conversion and 78% selectivity at 25 °C. When the temperature rises to 40 or 50 °C, there is remarkable improvement in conversion and selectivity, but the reaction at 50 °C shows the better catalytic efficiency. On the basis of these results, we concluded that the higher temperature can cause the higher conversion and selectivity. To the best of our knowledge, the high temperature results in improvement of the effective collision frequency, which also promote the reactivity in this process.

● Effect of the loading of hydrogen peroxide

It can be observed from Table 2 (entries 4, 7, 8, and 9) that the loading of H_2_O_2_ significantly affects the activity and selectivity of the reaction. When the loading of H_2_O_2_ was 0.25 or 0.5 mmol, the product showed sulfoxides preference but low conversion. With the increase of the addition of hydrogen peroxide from 1 mmol to 1.25 mmol, although the improvement of conversion is a little, the selectivity has significantly increased. Probably, the more hydrogen peroxide can generate more active species that play an important role in this reaction.

#### 2.2.2. Control Experiment and Scope in Various Sulfides

The control test (Table S1) implied that the reaction occurred little without using catalyst as NiCl_2_·6H_2_O and HEDP had little reactivity. Therefore, we speculated that the POMs units may play an important role in catalytic process [37].

Afterwards, the scope of experiment was investigated with other substrates, as shown in Table 3. The results of using **1** to oxidize these sulfides (Table 3, entry 1–7) in the optimal conditions have exhibited variable reactivity of sulfides substrates: methyl ethyl sulfide and dipropyl sulfide were oxidized with 100% conversion and 100% selectivity (Table 3, entry 1 and 2), and n-butyl sulfide gave 100% conversion and 97% selectivity (Table 3, entry 3). Methyl *p*-tolyl sulfide is less active with 99% conversion and 94% selectivity (Table 3, entry 4), while ethyl phenyl sulfide showed 97% conversion and 94% selectivity (Table 3, entry 5). Moreover, it is important to highlight that inert diphenyl sulfide and dibenzothiophene (0.5 mmol) were almost completely oxidized to sulfone with 98% conversion and 93% selectivity (Table 3, entry 7), 99% conversion and 99% selectivity (Table 3, entry 8), respectively. In general, the results and the reactivity of sulfides we obtained are in the sequence alkyl–alkyl thioethers (Table 3, entry 1–3) > aryl–alkyl thioethers (Table 3, entry 4–6) > aryl–aryl thioethers (Table 3, entry 7 and 8). The reactivity lowers due to steric hindrance of the reactants. But, 1-methoxy-4-methylsulfanylbenzene has displayed the excellent reactivity for the formation of 100% sulfone, mainly due to the electron-donating groups on the aromatic ring accelerates transformation of sulfides to oxygenated products [30].

On the basis of our findings, it can be concluded that steric hindrance and electronic effect play an important role for the transformation of sulfides to sulfones: the less steric hindrance or electron-donating groups on the aromatic ring in sulfides can lead to the higher conversion and selectivity. Notably, the admirable conversion and selectivity of dibenzothiophene may be potentially useful for oxidative desulfurization in petrochemical. In addition, there is comparative data (Table S2) of previously-reported catalysts and the catalyst used in this work for oxidation of thioanisole. As described, compound **1** has demonstrated the better reactivity and fast reaction rate compared to the previous reports.

#### 2.2.3. Recycling Experiment

**Figure 2:**
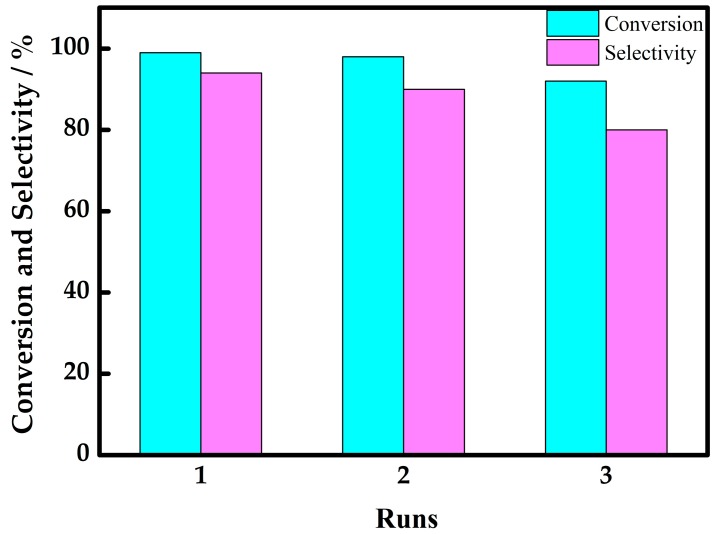
The conversion and selectivity histogram of every recycle.

The recyclability of the catalyst was investigated in the oxidation of thioanisole under the optimal conditions (**1**: 25 mg; thioanisole: 0.5 mmol; 50 °C; hydrogen peroxide: 1.25 mmol; acetonitrile: 5 mL). At the end of every reaction, the catalyst can be easily recovered from the solvent/oxidant/substrate system by filtration, after that organic compounds were removed completely leaving catalyst in the tube, further washed three times by acetonitrile, the mixture was dried in oven at 60 °C. The infrared spectra of compound 1 after reaction completion was used to analyze catalyst composition, and then duplicate test proceeded with the unchanged catalyst. The results shown in Figure S5 indicate no obvious changes in catalyst during the three runs. The histogram in Figure 2 showed catalytic activity had no distinct loss of initial catalytic activity, which was observed after three-runs of duplicate operations. Unfortunately, the infrared spectra confirmed that the catalyst has changed just after the third run which may be due to the collapse of the POMs skeleton. The corresponding conversion and selectivity of the fourth run (Table S3) has changed obviously.

### 2.3. TGA

The thermal stability of compounds **1** and **2** have been investigated in flowing N_2_ atmosphere with heating at a speed of 10 °C min^−1^ in the temperature range 25–800 °C.

**Figure 3:**
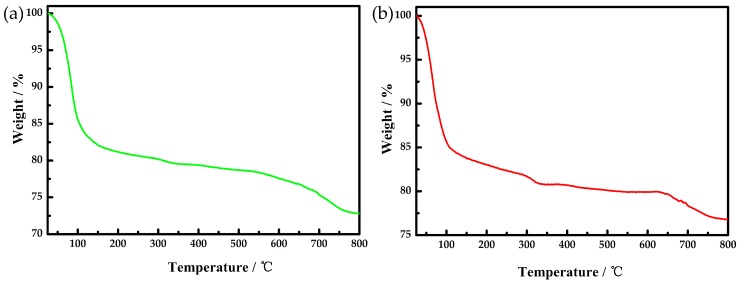
(**a**) The TGA curve of compound **1**; (**b**) The TGA curve of compound **2**.

The TGA curve of **1** shown in Figure 3 exhibits only one step weight loss in the temperature range 25–800 °C. The total weight loss is 26.71% corresponding to the 20 crystal water molecules and organic moieties in **1** (26.40%). There were six efflorescent crystal water molecules in **1**.

The TGA curve of **2** in Figure 3 showed one successive weight loss step in the temperature range 25–792 °C. The total weight loss is 23.33%, corresponding to the 17 crystal water molecules and organic moieties in **2** (23.71%). There were seven efflorescent crystal water molecules in **2**.

### 2.4. UV Spectra

**Figure 4:**
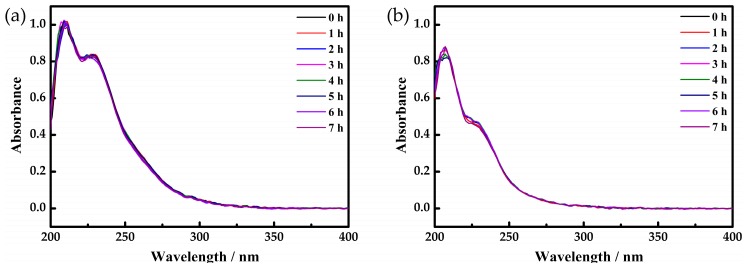
UV spectra of compounds **1** (**a**) and **2** (**b**).

As shown in Figure 4, the UV spectra of **1** and **2** are monitored in the range of 200–400 nm. The strong peak at 208 nm is assigned to charge transfer transition of pπ-dπ from O_t_ to Mo, while the characteristic absorption peak around 228 nm can be attributed to the charge transfer of pπ-dπ from O_b_ to Mo. In order to investigate the stability of **1** in solution, systematic studies of UV-vis spectra for time-dependent were monitored. As it has described in Figure 4, both two compounds remain stable for at least 7 h at room temperature. As a result, UV absorption peaks of two compounds are almost unchanged in principle, which proves that they can exist stably in aqueous solution for at least seven hours.

### 2.5. Solution ^31^P NMR Studies

We studied the solution ^31^P NMR spectra (D_2_O) of **1** and **2** at room temperature. The chemical shifts of the solution^31^P NMR spectra were shown in Table S4 for two POMs and HEDP (referenced to 85% H_3_PO_4_).

**Figure 5:**
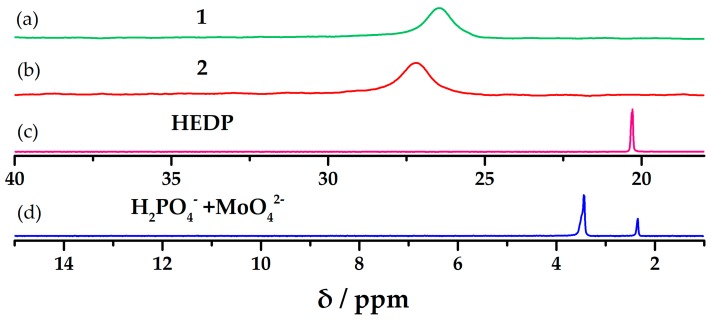
(**a**) solution ^31^P NMR spectra of **1**; (**b**) solution ^31^P NMR spectra of **2**; (**c**) Solution ^31^P NMR spectra of 1-hydroxy ethidene diphosphonic acid (HEDP); (**d**) Solution ^31^P NMR spectra of the physical mixture of phosphate and molybdate. ^31^P NMR spectra appear at 0 ppm were referred to 85% H_3_PO_4_.

As shown in Figure 5, the ^31^P NMR spectra have shown that there is only one signal for each compound, namely 26.428, 27.148, and 22.196 for **1**, **2**, HEDP, and the physical mixture of phosphate and molybdate, respectively. Moreover, from the results we obtained that all P atoms in every compound are magnetically equivalent phosphorus corresponding to the symmetrical structure. Although all P atoms in two POMs belonged to the HEDP in POMs framework, there were few differences in the ^31^P NMR chemical shifts of three compounds, which are ascribed to the influence of transition metals.

**Figure 6:**
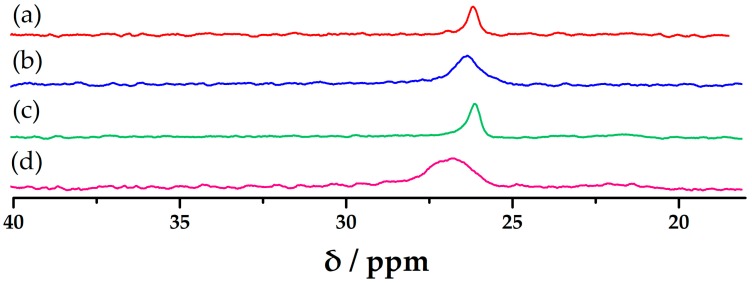
Solution ^31^P NMR spectra of **1** after different tests. (**a**) **1** in CH_3_CN after 4 h at 50 °C; (**b**) **1** in CH_3_CN after five days at room temperature; (**c**) 30 mg of **1** after the second catalytic test in optimal conditions; (**d**) 30 mg of **1** after the third catalytic test in optimal conditions; ^31^P NMR spectra appears at 0 ppm were referred to 85% H_3_PO_4_.

To investigate the stabilization of **1** for catalysis, it was dried in vacuum oven after the tests in different conditions, and then the dried samples were dissolved in D_2_O to get the solution ^31^P NMR spectrum. As shown in Figure 6, the results revealed that **1** had no obvious changes in various conditions except it went through three cyclic tests. Therefore, **1** is stable during the catalytic process.

## 3. Materials and Methods

### 3.1. Materials

All of the reagents used were of analytical grade and obtained from commercial sources without further purification. Na_2_HPMo_12_O_40_·14H_2_O was prepared using literature methods and characterized by IR spectroscopy (Nicolet, Madison, WI, USA) [38]. All of the the sulfides were purchased from J&K Chemical (Beijing, China). Hydrogen peroxide was obtained from Deen Reagent (Tianjin, China).

#### Synthesis of Catalysts

● Synthesis of Na_8_[NiO_2_{Mo_2_O_5_(O_3_PCH_3_C(O)PO_3_)}_2_]·26H_2_O (**1**)

The synthesis of compound **1** performed via a one pot reaction that using aqueous solution (10 mL) containing Na_2_HPMo_12_O_40_·14H_2_O (2.12 g, 1.0 mmol), HEDP (0.41 g, 1.99 mmol), NiCl_2_·6H_2_O (0.24 g, 1.01 mmol) and 0.4 mL 0.25 mol·L^−1^ tetrabutylammonium bromide aqueous solution that was alkalized with 12 mol·L^−1^ NaOH to adjust the pH to 6.7. After stirring at 90 °C for 2 h, the precipitate was removed by filtration. The light green strip shape crystals of **1** were collected after about three weeks. Elemental analysis (%) calcd for **1**: C, 2.84; H, 3.43; Ni, 3.47; Mo, 22.72. Found: C, 2.99; H, 3.56; Ni, 3.51; Mo, 23.18. IR (KBr, cm^−1^): 3433 (br), 2969 (w), 2871 (w), 1148 (s), 1115 (s), 1068 (s), 1041 (s), 973 (s), 918 (s), 892 (m), 864 (m), 851 (m), 749 (m), 721 (w), 622 (w), 523 (w).

● Synthesis of Na_8_[CoO_2_{Mo_2_O_5_(O_3_PCH_3_C(O)PO_3_)}_2_]·24H_2_O (**2**)

The preparation of **2** (red block shape crystals) is quite similar to 1, but with CoCl_2_·6H_2_O (0.24 g, 1.01 mmol) instead of NiCl_2_·6H_2_O and the pH was adjusted to 6.8. Elemental analysis (%) calcd for **2**: C, 2.90; H, 3.27; Co, 3.56; Mo, 23.22. Found: C, 3.12; H, 3.10; Co, 3.46; Mo, 23.45. IR (KBr, cm^−1^): 3439 (br), 2970 (w), 2870 (w), 1143 (s), 1117 (s), 1069 (s), 1042 (s), 972 (s), 917 (s), 891 (s), 856 (m), 745 (m), 723 (w), 619 (w), 536 (m).

### 3.2. Characterization

Elemental analyses of C, H, and N were performed with an Elementar Vario Elcube CHNS analyzer (Perkin-Elmer, Waltham, MA, USA). Elemental analysis for Mo, Co and Ni were performed with a Perkin Eimer Optima 2100 DV (Perkin-Elmer, Waltham, MA, USA) inductively coupled plasma optical emission spectrometer. IR spectra were recorded on a Bruker VERTEX 70 IR spectrometer (Nicolet, Madison, WI, USA) (using KBr pellets) in the range of 4000–400 cm^−1^. X-ray powder diffraction (XRPD) data were recorded on a Bruker AXS D8 Advance diffractometer (Bruker, Karlsruhe, Germany) with Cu Kα radiation in the angular range 2θ = 5°–45° at 293 K. TG analysis was measured on NETZSCH STA449F5/QMS403D instrument (Mettler-Toledo, Schwerzenbach, Switzerland) with a heating rate of 10 °C min^−1^ from 25 °C to 800 °C in N_2_ flow. The solution ^31^P NMR spectra were detected in 5 mm tubes on a Bruker AV-400 model spectrometer (Bruker, Karlsruhe, Germany) operating at 400 MHz. ^31^P chemical shifts were referenced to 85% H_3_PO_4_ as the external standard.

## 4. Conclusions

In summary, two sandwich-type POMs were synthesized by one-pot reaction, which have shown excellent catalysis for oxidization of thioanisole. Besides this, compound **1** exhibited splendid catalytic activity and a high selectivity of oxidizing various sulfides to sulfones. In this work, we have discussed those factors that affect the reaction rate in different conditions or with various sulfides. From the results, one can conclude that little steric hindrance and electron-donating groups on the aromatic ring are beneficial for the oxidative process. Furthermore, good recyclability, superior thermal, and hydrolytic stability imply that the POMs have the potential in catalytic oxidation of organic sulfide. Additionally, POMs can be modified by different functional groups at the molecular level to endow their distinct properties, which is in good consistency with the results in this work. Moreover, future work will focus on the regulation of POMs at molecular level to obtain unexceptionable catalysts and the characterization of further mechanistic studies.

## Figures and Tables

**Table 1:** Crystal data and structure refinements for the compounds **1** and **2**.

Compounds	1	2
Formula	C_4_H_58_Mo_4_Na_8_NiO_52_P_4_	C_4_H_54_CoMo_4_Na_8_O_50_P_4_
Formula weight/(g mol^−1^)	1688.77	1652.96
T (K)	293(2)	296(2)
Crystal system	triclinic	monoclinic
Space group	*P*-1	*C2/c*
a/Å	9.2505(9)	23.5940(16)
b/Å	10.8049(11)	9.8626(6)
c/Å	14.3226(13)	21.1105(14)
α/°	84.259(2)	90
β/°	84.085(2)	90.8400(10)
γ/°	65.2980(10)	90
Volume/(Å^3^)	1291.1(2)	4911.8(6)
Z	1	4
D_calc__d_ (g cm^−3^)	2.172	2.235
μ/(mm^−1^)	1.620	1.652
F(000)	842.0	3284.0
Crystal size/(mm^3^)	0.5 × 0.22 × 0.2	0.55 × 0.55 × 0.45
Radiation	MoKα (λ = 0.71073)	MoKα (λ = 0.71073)
2Θ range for data collection/°	4.924 to 50.2	3.86 to 50.198
Limiting indices	−10 ≤ h ≤ 11, −10 ≤ k ≤ 12, −17 ≤ l ≤ 15	−28 ≤ h ≤ 26, −11 ≤ k ≤ 11, −25 ≤ l ≤ 19
No. of reflections collected	6591	11983
No. of independent reflections	4519 {Rint = 0.0171, Rsigma = 0.0315}	4356 {Rint = 0.0163, Rsigma = 0.0196}
No. of parameters	337	322
GOF on *F*^2^	1.091	1.080
R_1_, wR_2_ [*I* > 2σ(*I*)]	0.0276, 0.0725	0.0291, 0.0776
R_1_, wR_2_ [all data]	0.0310, 0.0745	0.0310, 0.0786

**Table 2:** Results for catalytic oxidation of thioanisole by compound **1** with H_2_O_2_ in acetonitrile in different conditions after 1 h.

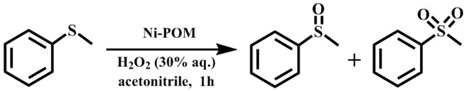						
**Entry**	**Amount of Catalyst (mol %) ^a^**	**Temp. (°C)**	**H_2_O_2_ (mmol)**	**Conv. (%)**	**Selectivity (%)** **Sulfoxide/Sulfone**	
1	None	50	1.25	37	16	84
2	1	50	1.25	90	20	80
3	2	50	1.25	96	11	89
4	3	50	1.25	98	6	94
5	3	25	1.25	63	22	78
6	3	40	1.25	92	19	81
7	3	50	1	96	27	73
8	3	50	0.5	47	79	21
9	3	50	0.25	24	88	12

Reaction conditions: thioanisole (0.5 mM, 0.06 mL); acetonitrile (5 mL). All products were identified by GC–MS spectra. The results refer to GC spectra based on dodecane as internal standard. ^a^ mol % = [*n*_(catalyst)_/*n*_(thioanisole)_] × 100%

**Table 3:** Oxidation of various sulfides with H_2_O_2_ in the presence of the catalyst in acetonitrile.

Entry	Substrate	Temp./°C	Time/h	Conv./%	Selectivity (%)	
					Sulfoxide/Sulfone	
1 ^a^	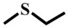	50	0.5	100	0	100
2	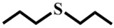	50	1	100	0	100
3	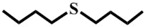	50	1	100	3	97
4	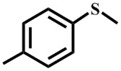	50	1	99	6	94
5	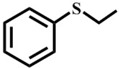	50	1	97	6	94
6	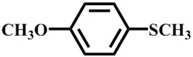	50	1	100	0	100
7 ^b^	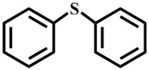	60	1	98	7	93
8	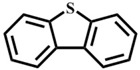	60	3	99	0.8	99

^a^ Reaction condition for the entries 1 to 6: catalyst, 3 mol %; substrate, 0.5 mmol; acetonitrile, 5 mL; H_2_O_2_, 1 mmol. ^b^ Reaction condition for entries 7 and 8: catalyst, 3 mol %; substrate, 0.5 mmol; acetonitrile, 2.5 mL; H_2_O_2_, 1.5 mmol. All of the products were identified by GC–MS spectra. The results refer to GC spectra based on dodecane as internal standard.

## Supplementary Materials

The following figures and tables in Supplementary Materials are available online at www.mdpi.com/1996-1944/10/10/1173/s1, Figure S1: The XRPD patterns of **1** and **2**; Figure S2: The IR spectra of two compounds; Figures S3 and S4: The representation of the synthesis of two POMs; Figure S5: The infrared spectra of catalyst after the reactions. Table S1: The results of control tests; Table S2: the results that 1 compared with other catalysts; Table S3: The conversion and selectivity of catalytic reaction after cyclic tests. Table S4: Solution ^31^P NMR chemical shifts of 1, 2 and HEDP.
